# Entrepreneurs’ Capacity for Mentalizing: Its Influence on Burnout Syndrome

**DOI:** 10.3390/ijerph18010003

**Published:** 2020-12-22

**Authors:** Guadalupe Manzano-García, Juan Carlos Ayala-Calvo, Pascale Desrumaux

**Affiliations:** 1 Department of Education Sciences, Universidad de La Rioja. C/La Cigüeña, 60, 26004 Logroño, Spain; 2 Department of Economics and Business, Universidad de La Rioja. C/La Cigüeña, 60, 26004 Logroño, Spain; juan-carlos.ayala@unirioja.es; 3 Laboratory EA. 4072 PSITEC, Univ. Lille, France. Department of Psychology, BP 60149, F-59655 Villeneuve d’Ascq Cedex, France; pascale.desrumaux@univ-lille3.fr

**Keywords:** entrepreneur, entrepreneurial burnout, emotional exhaustion, hypermentalizing, hypomentalizing, mentalizing

## Abstract

Burnout is a mental disorder that leads to difficulties for the entrepreneur in controlling his or her personal and professional life. The most common consequences of entrepreneurial burnout include the subject experiencing low motivation, low organizational commitment, loss of energy, demoralization in connection with their work, poor quality of work, feeling of failure, and the perception that his or her company is performing poorly. We used a sample of 157 Spanish entrepreneurs selected at random from the Iberian Balance Sheet Analysis System database. We employed the Spanish version of the Reflective Functioning Questionnaire to measure mentalizing and the Spanish version of the Maslach-Burnout Inventory-General Survey (MBI-GS) to measure burnout. This research showed that entrepreneurial burnout could be avoided in part if the entrepreneur achieved a good capacity for mentalizing. Hypomentalizing contributed to explaining entrepreneurs’ levels of professional efficacy, cynicism, and emotional exhaustion. In contrast, the explanatory power of hypermentalizing was not significant for any of the dimensions of burnout. This study provides new evidence of burnout in entrepreneurs; a professional group with an important economic, politic, and social role has been little studied.

## 1. Introduction

In an increasingly complex, changing, and unpredictable environment, entrepreneurs are considered important drivers of economic growth and countries’ social, technical, and economic development. At the root of this statement is recognizing the important role that entrepreneurs play in creating new jobs, exploiting new employment opportunities, designing and developing new products and services, and stimulating competition and competitiveness [1,2]. 

Entrepreneurial activities are usually associated with a high degree of ambiguity in terms of results, as normally, the information available for decision-making is incomplete or ambiguous. In addition, entrepreneurs often have long and intense workdays to ensure that their company is maintained in the market and achieve established objectives. These circumstances, among others, make the appearance of burnout syndrome more likely [3]. In research on entrepreneurship, the most accepted definition of burnout conceptualizes this syndrome as a process of progressive and continuous accumulation of chronic stress, characterized by emotional exhaustion, cynicism, and lack of professional efficacy, which appears among those professionally involved with others [4]. Emotional exhaustion refers to the depletion of emotional resources in response to a highly professional demand from the environment. The subject experiences a sense of emptiness and not being able to give more of themselves [5]. Cynicism refers to the lack of interest in what is happening in the environment, the deterioration of interpersonal relationships, and the maintenance of a negative attitude towards employees and clients [6]. Lack of professional efficacy reflects the entrepreneur’s low self-esteem and guilt that their social skills and ability to do their job well have diminished [7].

The World Health Organisation has included “burnout” in the International Classification of Diseases (ICD) that will come into force in 2022. In that classification, burnout is defined as a mental illness that leads to difficulties controlling their own personal and professional life [8]. This syndrome affects both the behavior of entrepreneurs and the future of their companies. An entrepreneur’s negative emotions can leave them burnt out and lead them to experience negative thoughts about their ability to direct the company [6] effectively. The most common consequences of entrepreneurial burnout include the subject experiencing low motivation, low organizational commitment, loss of energy, demoralization in connection with their work, poor quality of work, feeling of failure, and the perception that his or her company is performing poorly [9,10,11]. 

From the perspective of the Theory of the Mind [12], burnout could be partly avoided if entrepreneurs had good mentalizing. The concept of mentalizing refers to how the individual explains their behavior and that of others through the interpretation the subject makes of the reasons, causes, and motives of an event [13]. This type of attribution may be correct or incorrect and may influence the degree of the entrepreneur’s burnout.

Mentalizing “is a form of social cognition” [14] that allows individuals to understand their behavior and that of others, which is often intuitive and emotional; it represents the ability to regulate emotions and to be able to create positive and satisfactory interpersonal relationships. Emotions organize perceptions and thoughts [15]. Mentalizing allows one to give meaning to their internal experience and the external world; it facilitates satisfactory interpersonal functioning where the person feels connected with others while managing to maintain their sense of individuality [16].

Mentalizing implies interdependence between the subjective knowledge of the mental states of oneself and others; it influences our behavior [17], allows us to feel that we control our thinking and way of acting, and how we perceive, process, and interpret social cues in the environment. In the process of mentalizing, we can distinguish two abnormal ways of interpreting signals from the environment (hypomentalizing and hypermentalizing) that allow the type of error identified in this interpretation to be classified. Hypomentalizing or a propensity to infer less social meaning makes our own and other mental processes more difficult. This error makes understanding how actions are detrimental to others more difficult [12], which makes one think about the relevance of the capacity for mentalizing in the operation of entrepreneurs. Hypermentalizing is the propensity to over-attribute without possessing a knowledge of objective reality. Therefore, hypermentalizing would be an “over-interpretation of the mental states of others, which leads to misunderstandings and disables a stable development of interpersonal relations…” [18]. From a theoretical point of view, it would be appropriate for the entrepreneur to show very low levels of both hypomentalizing and hypermentalizing (almost zero) [19].

Being lucid, being curious, trying new things, being able to decide in uncertain environments, or having the courage to fail are attitudes that are at the heart of mentalizing and are of great importance in the world of the entrepreneur [20,21]. Mentalizing refers to the knowledge structures that people use to evaluate, judge, or decide anything; it involves identifying and evaluating opportunities, creating, and developing companies [22]. Biases in the capacity for mentalizing involve different types of behavior. For example, the fear of missing an opportunity leads to action bias, which can lead the entrepreneur to accelerate the process of starting a business. Fear of failure tends to generate biases in analysis and planning [23].

The capacity for mentalizing is a pillar of mental health [20]. Alterations in the mentalizing process led entrepreneurs to misinterpretations that generate psychological discomfort, alterations in behavior, and interpersonal relations. An entrepreneur with a low mentalizing capacity will be worse at tolerating the pressures and possible changes that may occur in the environment, which may contribute to the appearance of burnout syndrome.

The concepts of burnout and mentalizing are complex multidimensional constructs. Both are related to motivations, beliefs, intentions, reasons, desires, needs, a deficit of social and cognitive skills, etc. [5,6,24]. Both burnout syndrome and mentalizing are defined as a process and therefore change over time. There are no studies that have analyzed the relationship between these two concepts in samples of entrepreneurs. Therefore, this paper’s main objective was to determine the extent to which mentalizing capacity may explain the degree of burnout suffered by entrepreneurs. More specifically, we tried to determine how the dimensions of mentalizing (hypomentalizing and hypermentalizing) may influence the dimensions of burnout (professional inefficacy, cynicism, and emotional exhaustion). This study contributes to describing the relationship between the capacity of mentalizing and burnout. It also provides new evidence of burnout in entrepreneurs; a professional group that has an important economic, politic and social role has been little studied up to now.

## 2. Materials and Methods

### 2.1. Participants 

The participants were 157 entrepreneurs, manager-owners of Spanish private companies that were active in January 2019 and had a workforce of over ten people and fewer than 50 (Small Businesses). The participants’ age range was from 27 to 70 years (*M* = 48.38; standard deviation (SD) = 8.65). The majority of the participants were men (70.7%). In terms of the highest education level, 8.9% finished at primary level, 29.9% at secondary, and 61.1% at higher studies. Respondents who lived with their partner were 92.4% and those who lived alone were 7.6%. On average, the number of children they had was 1.53 (*SD* = 0.94; range: 0–4 children). In terms of work, 45.9% of entrepreneurs worked in the services/transport sector, 12.1% worked in construction, 31.2% worked in industry, and 10.8% in food. The companies’ average number of employees was 27.39 (*SD* = 14.70; range: 10–50 employees) and the hours worked per week were 49.17 (*SD* = 10.79; range: 25–70 h). All participants had given their informed consent for inclusion before they participated in the study. The study was conducted in accordance with the Declaration of Helsinki and approved by the ethics committee at the Public University of La Rioja (code CE-10-2020).

### 2.2. Measures

We used the Spanish version [25] of the Reflective Functioning Questionnaire (RFQ-8) [26] to measure mentalizing. We used the short version, recommended for research and to avoid respondents’ fatigue. The instrument consists of 8 items: 4 measure hypermentalizing (e.g., “People’s thoughts are a mystery to me”) and 4 measure hypomentalizing (e.g., “When I get angry I say things that I regret”). The instrument uses a Likert-type scale ranging from 1 “strongly disagree” to 7 “strongly agree”. High scores on both scales reflected difficulties in reflective capacity. Average scores would reflect adequate reflection capacity (genuine). The Reflective Functioning Questionnaire (RFQ-8), as well as its translated versions, have proven their reliability and validity in several validation studies [26,27,28,29]. In this investigation, Cronbach’s alpha for hypermentalizing and hypomentalizing were 0.86 and 0.80, respectively.

We used the Spanish version [30] of the Maslach-Burnout Inventory General Survey (MBI-GS) [31] to measure burnout. The instrument consists of 16 items. The subscale of emotional exhaustion (EE) consists of 5 items (e.g., “I am emotionally exhausted by my work”). The subscale of cynicism (C) includes 5 items (e.g., “I’ve become more cynical about the usefulness of my work”). The subscale of professional efficacy (PE) covers 6 items (e.g., “I can effectively solve the problems that arise in my work”). The entrepreneurs valued each item by making use of a Likert-type scale that ranges from 0 “never” to 6 “always”. In this investigation, Cronbach’s alpha coefficients were 0.90 for emotional exhaustion, 0.87 for cynicism and 0.91 for professional efficacy. 

Based on the previous literature on burnout, we included the number of children, level of education, gender, marital status, age, number of employees, working hours per week, and the sector of activity as control variables [10].

### 2.3. Design and Procedure

Data were collected through an online questionnaire using the collaborative software Google Forms. An email containing a brief explanation of the study’s objectives was sent by the researchers, along with a link to the questionnaire. All procedures followed in this work were in accordance with the responsible committee’s ethical standards on human experimentation (institutional and national) and with the Helsinki Declaration of 1975, as revised in 2000. Informed consent was the first question on the online questionnaire. It was not possible to continue completing the online questionnaire until informed consent was accepted. Two reminders were sent to increase the response rate: the first was sent 7 days after the initial email and the second was sent 15 days after the initial email. Online self-reports have the same validity and reliability as those carried out conventionally [32]. 

The sample needed for this study, calculated with the help of the G* Power v.3.1.9.2 [33], taking into account an effect size (f^2^) of 0.15 and a statistical significance level of 0.05, was 138 observations [34]. The entrepreneurs were randomly selected from companies in the Iberian Balance Sheet Analysis System database that met the inclusion criteria. The participants were volunteers. They were surveyed twice in 2019. In June 2019 (T1), the data collected was related to mentalizing. In September 2019 (T2), the data obtained was on burnout. This was done to avoid common method variance.

Of the 1100 online questionnaires sent out on T1, 231 returned useable questionnaires (21%). Of the 231 questionnaires sent out on T2, 160 were returned, although 3 were discarded as they were incomplete (response rate = 69.3%). The final sample was composed of 157 entrepreneurs, representing a statistical power of 97.38%. When we compared the final sample with the sample of lost subjects, there were no statistically significant differences between the mean values of the burnout and mentalizing dimensions. 

### 2.4. Data Analysis

We used hierarchical linear regression analysis to determine the role of mentalizing factors in explaining each of the dimensions of burnout. In these analyses, the dimensions of burnout (professional efficacy, cynicism, and emotional exhaustion) were the outcome variables, and the explanatory variables were the two dimensions of mentalizing (hypermentalizing and hypomentalizing). The three models’ validity was evaluated according to the *R*^2^, the corrected *R*^2,^ and the F-test of statistical significance. All analyses were performed with the SPSS Statistics software, version 26.0 (IBMCorp, Armonk, NY, USA) [35].

Before the online questionnaires were sent, a preliminary investigation was carried out with 35 entrepreneurs who lived in the same region as the researchers. All of them decided to participate voluntarily. The results of these questionnaires were not included in further analyses. The study aimed to confirm the items’ clarity and the validity of the content of the scales used in the study. The pilot study’s participants concluded that the clarity of the items was good. The measurement instruments used were in Spanish and demonstrated adequate psychometric properties. To reduce the risk of bias in the analysis, it was decided that questionnaires that were incomplete or showed repeated options in one scale were excluded.

## 3. Results

The correlations between the variables used in the work, their mean values, and their standard deviations can be seen in Table 1. The results confirm that there is discriminant validity since all correlations are lower than 0.58, below the threshold of 0.85 established by some authors as the cut-off point [36].

The entrepreneurs in the sample possess, on average, low levels of burnout: high levels of professional efficacy, together with low levels of emotional exhaustion and cynicism [37]. These burnout levels are slightly lower than those found by De Mol et al. [7] in a sample of 326 entrepreneurs who were members of the 39 Business Networking International (BNI) groups in the Virginia Region; or those found by Soenen et al. [11], who measured the emotional exhaustion (considered the key dimension of burnout) of 236 entrepreneurs collected from a national sample of entrepreneurs located in continental France.

The results showed that hypomentalizing negatively correlates with professional efficacy and positively with cynicism and emotional exhaustion. On the contrary, hypermentalizing correlates positively with professional efficacy and negatively with cynicism and emotional exhaustion. The biggest correlation between mentalizing factors and burnout was between hypomentalizing-cynicism-and hypermentalizing-cynicism. These findings show, on the one hand, that entrepreneurs who hypomentalizing will be more likely to suffer burnout than those who hypermentalizing; on the other hand, that deficiencies in the capacity for mentalizing affect above all the dimension of burnout, which refers to cynicism, the detachment of the entrepreneur from his environment, the tendency to isolate themselves, and to show negative attitudes towards others [6].

Table 2 shows the results of the regression analysis. The values of the Durbin-Watson statistic ranged from 1.5 to 2.5 (acceptance range). Thus, we can say that there were no autocorrelation problems. On the other hand, the condition index values ranged from 15 to 20 (acceptance range), and all the variance inflation factor (VIF) values stayed below 5. Thus, we can say that there were no multicollinearity problems.

In the models in which the outcome variable was professional efficacy and emotional exhaustion, control variables explained 9% and 8% of the variance, respectively (*p* 0.01). In both cases, *R*^2^ increased by 12% when mentalizing factors were introduced. In the model in which the outcome variable was cynicism, control variables explained 20% of the variance. When mentalizing factors were introduced, the increase in *R*^2^ was 15%. Moreover, the results showed that hypomentalizing contributes to explaining entrepreneurs’ levels of professional efficacy, cynicism, and emotional exhaustion. In contrast, the explanatory power of hypermentalizing was not significant for any of the dimensions of burnout. 

The *R*^2^ value of each of the models showed values above 0.24, and the adjusted *R*^2^ was above 0.19. Although we cannot compare these values with those found in previous studies, considering that we are working with variables that attempt to measure human behavior, the resultant *R*^2^ values are considered acceptable for this research area [34]. We should also consider that *R*^2^ provides an estimate of the strength of the relationship between the predictor variables and the dependent variable, but does not provide a formal hypothesis test for this relationship. The test that determines whether this relationship is statistically significant is the F test of statistical significance. In the three models analyzed, the F value was significant (*p* 0.001).

## 4. Discussion 

In a sample of entrepreneurs, this work examined the role of mentalizing in explaining burnout syndrome. As far as we are aware, the relation between these factors has not been researched previously.

The results we obtained confirm that hypomentalizing contributes to explaining each of the burnout dimensions, although with different weighted values. More specifically, the value of the coefficient *β* was positive in the models of emotional exhaustion (*β* = 0.34) and cynicism (*β* = 0.33), while its value was negative for the professional efficacy aspect (*β* = −0.28). These results indicate that the greater the degree of hypomentalizing in an entrepreneur, the greater the burnout level. 

Entrepreneurs who have a propensity to infer less social meaning have trouble understanding how their actions and attitudes can negatively affect others’ feelings [12], which leads to a decrease in both mental and psychological health [38]. In addition, entrepreneurs who are hypomentalizers have difficulties interpreting their own behavior and that of others; they tend not to judge behavior against an objective set of data but rather by "guessing" others’ ideas, beliefs, feelings, attitudes, or desires that underlie certain behaviors observed in certain contexts. This tends to lead to interpersonal relations that are not very positive or satisfactory. Our results support previous studies that showed that a deterioration in interpersonal relations generates dehumanization of professional and personal relationships and contributes to the emergence of burnout [9,11].

Mentalizing refers to the control of interpersonal communication and its social and emotional power. Successful entrepreneurs tend to be empathetic and cultivate good communication with their workers to transmit the values of their business. An entrepreneur who is a hypomentalizer usually has trouble correctly relating to professional environments. Thus, relationships with others could generate burnout [39]. In this sense, our results seem to support the arguments of those who have shown that the relationship between the entrepreneur and their subordinates and those they work with daily could be a factor explaining burnout [40].

According to our results, hypermentalizing does not contribute to explaining any of the dimensions of burnout. This result may be connected to the fact that hypermentalizing is usually not a characteristic of entrepreneurs. They do not usually tend to make assumptions beyond the objective information they possess [41]. A good entrepreneur usually has an adequate command of interpersonal relationships that lead them to create cohesive and involved work teams. On the other hand, they tend to be empathetic, good communicators, not over-interpret others’ mental states, and value people for what they bring to the organization, not through assumptions on their beliefs or needs, which leads them to assert their leadership [42,43]. 

Correlation analysis and the sign of the beta coefficients (β) in the regression analyses indicate that the greater the entrepreneur’s hypermentalizing, the lower their burnout levels. The sample entrepreneurs have a level of hypermentalizing that is below the average level of the measurement scale [26]. This result makes us question whether there is a minimum level of hypermentalizing beyond which burnout’s influence is significant.

On the other hand, it could be that although hypermentalizing does not have a direct effect on burnout, it could play a moderating role in its explanation. Thus, for example, hypermentalizing could moderate and reinforce the negative relationship between the entrepreneur’s capacity to build and maintain social networks and burnout, or the effectiveness of coping strategies in reducing burnout. It should also be explored whether hypermentalizing could reduce the positive impact that some of the working environment stressors (e.g., working hours per week) have on entrepreneurs’ burnout [10]. 

The results also showed some interesting effects of control variables. Firstly, hours of work a week showed a positive relationship with emotional exhaustion and lack of professional efficacy. Long working hours, which are common amongst entrepreneurs, limit the amount of time available for other leisure activities and, in the long term, generate significant interference with family life. These two relationships have been identified as sources of burnout [3]. On the other hand, gender helps to explain cynicism and emotional exhaustion, and the level of studies achieved contributes to explaining entrepreneurs’ level of emotional exhaustion. Other researchers found that work-related burnout is significantly lower in men than women, and that significant differences exist in the burnout levels associated with work depending on the level of studies obtained [10].

### 4.1. Practical Implications

Bearing in mind the importance of entrepreneurs in the economic and social development of countries and regions across the world [2], and the economic resources that governments provide for promoting entrepreneurship [44], we believe that our findings have several practical implications. Firstly, entrepreneurs, who are often overly involved in their work, tend to forget the significant effect that their physical and mental health has on their company’s development [45]. Understanding the negative influence that hypomentalizing has on their level of burnout and the negative consequences this can have on their personal life and the development and survival of their business, may help entrepreneurs to understand better the importance of developing skills that allow them to communicate correctly with their environment (employees, clients, suppliers, etc.). The acquisition of cognitive skills such as the capacity of understanding one’s behavior and that of others, or the ability to regulate emotions and to be able to create positive and satisfactory interpersonal relationships (mentalizing), will help entrepreneurs manage their company more effectively [6]. In fact, some authors believe that these skills are a source of sustainable competitive advantages [46]. Secondly, a decrease in the entrepreneur’s level of burnout, a consequence of optimal mentalizing capacity, would help them experience increased motivation and increased organizational commitment, increasing their propensity to maintain the company instead of having to close it [10]. This means that policymakers creating policies that support entrepreneurs should insist on developing programs that increase entrepreneurs’ mentalizing capacity, both in the present and in the future. These programs should be implemented in business faculties and schools, and even in pre-university education [47]. 

### 4.2. Limitations

This research has some limitations. Firstly, this is a time-lagged study. The amount of time that passed between measuring mentalizing and burnout was explicitly included in the design of the analysis to create a separation in time, context, and psychology and minimize the potential effects of common variance error. However, a time-lagged design does not allow a cause-effect relationship between the variables analyzed to be determined. Longitudinal studies are needed in various groups to explore the existing relationship between mentalizing dimensions and burnout. Secondly, we used a sample of entrepreneurs in only one country. It would be interesting to conduct similar research in other countries to determine whether our findings are similar in different cultural settings. Despite these limitations, this work covers an existing gap in the literature, trying to explore and find out more about the possible links between the process of mentalizing and burnout syndrome in a sample of entrepreneurs. 

## 5. Conclusions

Burnout is a mental illness that makes it difficult for entrepreneurs to control their personal and professional life. The key conclusion of the present work is that the negative consequences of this mental disorder, on both the entrepreneur’s health and that of their company, can be reduced if the entrepreneur achieves a good mentalizing capacity. 

## Figures and Tables

**Table 1:** Mean (M), standard deviation (SD), and correlations between variables.

	M	SD	1	2	3	4	5	6	7	8	9	10	11	12	13
1. Gender	1.29	0.46	1												
2. Age	48.38	8.65	−0.03	1											
3. Level of education	2.52	0.66	0.13	−0.02	1.00										
4. Marital status	1.92	0.27	0.08	−0.10	0.01	1									
5. Children	1.54	0.94	−0.10	0.05	−0.07	0.24 **	1.00								
6. Sector of activity	2.07	1.10	−0.05	−0.02	0.11	0.02	−0.09	1							
7. Number of employees	27.46	14.86	−0.17 *	−0.13	0.20 *	−0.08	−0.11	0.46 **	1.00						
8. Working hours per week	49.17	10.79	−0.23 **	−0.05	−0.14	−0.25 **	0.13	−0.08	−0.06	1					
9. Hypermentalizing	1.26	0.84	0.00	0.02	0.05	0.10	0.03	0.14	0.41 **	0.01	(0.86)				
10.Hypomentalizing	0.29	0.36	0.10	−0.01	0.02	−0.10	−0.13	−0.11	−0.19 *	0.08	−0.58 **	(0.80)			
11. Emotional exhaustion	1.73	0.96	0.13	−0.05	−0.15	−0.07	−0.12	−0.09	−0.15	0.23 **	−0.24 **	0.40 **	(0.90)		
12. Cynicism	1.32	1.05	−0.03	0.09	0.07	−0.12	−0.34 **	−0.15	−0.26 **	−0.02	−0.41 **	0.47 **	0.58 **	(0.87)	
13. Professional efficacy	4.44	1.00	−0.02	0.20 *	0.01	0.06	0.18 *	−0.11	−0.13	0.19*	0.22 **	−0.30 **	−0.26 **	−0.31 **	(0.91)

*M*: mean; *SD*: standard deviation. The Cronbach’s α of each of the scales are presented in parentheses on the diagonal, ** *p* 0.01; * *p* 0.05.

**Table 2:** Hierarchical linear regression of the relationship among burnout dimensions and mentalizing dimensions.

	Outcome Variable: Emotional Exhaustion						Outcome Variable: Cynicism						Outcome Variable: Professional Efficacy					
	Control Variables			Control Variables and Mentalizing			Control Variables			Control Variables and Mentalizing			Variables Control			Control Variables and Mentalizing		
	*β*	*t*	VIF	*β*	*t*	VIF	*β*	*t*	VIF	*β*	*t*	VIF	*β*	*t*	VIF	*β*	*t*	VIF
Gender	0.18 *	2.16	1.13	0.16 *	2.01	1.18	−0.14	−1.81	1.13	−0.15 *	−2.11	1.18	0.02	0.23	1.13	0.03	0.33	1.18
Age	−0.03	−0.40	1.05	−0.02	−0.23	1.07	0.06	0.83	1.05	0.09	1.28	1.07	0.21 **	2.68	1.05	0.19 **	2.55	1.07
Level of education	−0.13	−1.57	1.08	−0.15 *	−1.95	1.09	0.13	1.80	1.08	0.11	1.63	1.09	0.07	0.85	1.08	0.09	1.19	1.09
Marital status	0.02	0.19	1.20	0.04	0.55	1.24	−0.05	-0.58	1.20	0.00	−0.05	1.24	0.10	1.23	1.20	0.07	0.82	1.24
Children	−0.15	−1.88	1.13	−0.11	−1.37	1.16	−0.38 ***	−4.94	1.13	−0.33 ***	−4.72	1.16	0.11	1.30	1.13	0.06	0.83	1.16
Sector of activity	−0.02	−0.27	1.29	−0.02	−0.19	1.31	-0.06	-0.74	1.29	−0.06	−0.81	1.31	−0.07	−0.75	1.29	−0.06	−0.78	1.31
Employees	−0.08	−0.91	1.42	0.00	−0.02	1.78	−0.32 ***	−3.72	1.42	−0.19*	−2.22	1.78	−0.05	−0.57	1.42	−0.17	−1.71	1.78
Hours per week	0.26 ***	3.14	1.20	0.24 ***	2.94	1.24	0.02	−0.25	1.20	−0.04	−0.53	1.24	0.22 **	2.59	1.20	0.23 **	2.89	1.24
Hypermentalizing				−0.03	−0.32	1.93				−0.13	−1.40	1.93				0.12	1.16	1.93
Hypomentalizing				0.34 ***	3.66	1.64				0.33 ***	3.96	1.64				−0.28 ***	−3.02	1.64
R^2^	0.13			0.25			0.24			0.39			0.12			0.24		
R^2^ ajusted	0.09			0.20			0.20			0.35			0.08			0.19		
Change in F	2.84 **			11.22 ***			5.96 ***			18.12 ***			2.59 *			10.87 ***		

***t***: t-value; VIF: Variance inflation factor; *** *p* 0.001; ** *p* 0.01; * *p* 0.05. To facilitate reading, the significant coefficients have been written in bold.

## Data Availability

The data presented in this study are available on request from the corresponding author.
